# Genomic Insights Into Cadmium Resistance of a Newly Isolated, Plasmid-Free *Cellulomonas* sp. Strain Y8

**DOI:** 10.3389/fmicb.2021.784575

**Published:** 2022-01-28

**Authors:** Jinghao Chen, Likun Wang, Wenjun Li, Xin Zheng, Xiaofang Li

**Affiliations:** ^1^Hebei Key Laboratory of Soil Ecology, Center for Agricultural Resources Research, Institute of Genetics and Developmental Biology, Chinese Academy of Sciences, Shijiazhuang, China; ^2^University of Chinese Academy of Sciences, Beijing, China

**Keywords:** cadmium resistance, *Cellulomonas* sp., *zntA*, *copA*, gene expression, full genome

## Abstract

Our current knowledge on bacterial cadmium (Cd) resistance is mainly based on the functional exploration of specific Cd-resistance genes. In this study, we carried out a genomic study on Cd resistance of a newly isolated *Cellulomonas* strain with a MIC of 5 mM Cd. Full genome of the strain, with a genome size of 4.47 M bp and GC-content of 75.35%, was obtained through high-quality sequencing. Genome-wide annotations identified 54 heavy metal-related genes. Four potential Cd-resistance genes, namely *zntAY8*, *copAY8*, *HMTY8*, and *czcDY8*, were subjected to functional exploration. Quantitative PCR determination of *in vivo* expression showed that *zntAY8, copAY8*, and *HMTY8* were strongly Cd-inducible. Expression of the three inducible genes against time and Cd concentrations were further quantified. It is found that *zntAY8* responded more strongly to higher Cd concentrations, while expression of *copAY8* and *HMTY8* increased over time at lower Cd concentrations. Heterologous expression of the four genes in Cd-sensitive *Escherichia coli* led to different impacts on hosts’ Cd sorption, with an 87% reduction by *zntAY8* and a 3.7-fold increase by *HMTY8*. In conclusion, a Cd-resistant *Cellulomonas* sp. strain was isolated, whose genome harbors a diverse panel of metal-resistance genes. Cd resistance in the strain is not controlled by a dedicated gene alone, but by several gene systems collectively whose roles are probably time- and dose-dependent. The plasmid-free, high-GC strain Y8 may provide a platform for exploring heavy metal genomics of the *Cellulomonas* genus.

## Introduction

Microbial Cd resistance has been extensively studied in the past decades. A *Staphylococcus aureus* strain with plasmid-borne Cd resistance was first reported in 1968 (Sweeney and Cohen, 1968). Since then, a number of studies were conducted on bacterial species like *S. aureus*, *Cupriavidus metallidurans*, *Escherichia coli*, and *Bacillus subtilis* for Cd-resistance (Nies et al., 1989; Nucifora et al., 1989; Rensing et al., 1997; Solovieva and Entian, 2002). More recently, strains with superior Cd tolerance were isolated for various purpose (Baati et al., 2020; Kotoky and Pandey, 2020; Minari et al., 2020; Shi et al., 2020). For example, the *Cupriavidus* sp. strain WS2 has a minimal inhibitory concentration of 8 mM Cd (Shi et al., 2020), while that of the wild-type *E. coli* strain BL21 is below 1.2 mM (Qin et al., 2019).

Our current knowledge on genetic mechanisms of bacterial Cd tolerance is based on the exploration of specific resistance genes or operons like *cad*, *czc*, and z*nt* (Diels et al., 1995; Binet and Poole, 2000; Munkelt et al., 2004; Okkeri and Haltia, 2006; Monchy et al., 2007). All of them are found to play a vital role in the translocation/extrusion of intracellular Cd. They mainly fall into three categories including P-type ATPases, RND-driven efflux systems and cation diffusion facilitators (CDF; Nies, 2003). P-type ATPases and CDF transporters may function in transporting Cd from cytoplasm to periplasm (Paulsen and Saier, 1997; Busch and Saier, 2002; Saier et al., 2006; Scherer and Nies, 2009; Shamim et al., 2014), while RND-driven efflux systems such as CzcCBA probably export metals from periplasm to outside the cells (Legatzki et al., 2003; Stroebel et al., 2007). This two-step exporting mechanism by transporters of overlapping substrate specificity was commonly applied in the exporting of toxic substances in G^–^ bacteria (Tal and Schuldiner, 2009). In G^+^ bacteria where lack an outer membrane for the RND-driven efflux system to work, P-type ATPases are more common. Members of the P_IB_-family ATPases contain six to eight transmembrane (TM) helices, an ATP-binding domain and some strictly conserved motifs like the CPC motif (Arguello, 2003; Argüello et al., 2007; Smith et al., 2014). P_IB_-type ATPases can both transport monovalent cations such as Cu^+^ and Ag^+^ (e.g., CopA) (Sitsel et al., 2015; Purohit et al., 2018) and divalent cations such as Zn^2+^, Cd^2+^, and Pb^2+^ (e.g., CadA and ZntA) (Sharma et al., 2000; Wang et al., 2014; Sitsel et al., 2015). Some ATPases (e.g., CzcP) containing a conserved SPC motif are also known to transport Cd^2+^, Co^+^, Zn^2+^, Cu^+^, and Fe^2+^ (Scherer and Nies, 2009; Zielazinski et al., 2012; Smith et al., 2015; Patel et al., 2016). With the advent of the omics era (Methé and Lasa, 2013), there is a need to explore genetic systems for bacterial Cd resistance at the genomic level.

In this study, we aim to examine the genetic determinants for Cd resistance of a newly isolated *Cellulomonas* sp. strain Y8 at a genome-scale. A highly Cd-tolerant bacterial strain Y8 was isolated from a farmland soil and identified as a member of the *Cellulomonas* genus. Two Cd-resistant *Cellulomonas* sp. strains have been reported currently (Dell’Amico et al., 2005; Fouché, 2018) while little is known about their genetic mechanism. Metabolic potentials, Cd resistance and cell morphology were tested to characterize the strain. A high-quality full genome of strain Y8 was obtained through next-generation sequencing, based on which a genome-wide screening of metal-resistance genes were conducted. Four genes with Cd-resistance potential were subjected to quantitative PCR determination of *in vivo* expression in response to Cd stress, and heterologous expression in *E. coli* for functional verification. Of them, two potential P_IB–_type ATPases *zntA* and *copA* and an ACR3 family gene *HMT* were strongly Cd-inducible, but differentially expressed over time course and against Cd concentrations. Besides, *zntAY8* reduced *E. coli*’s intracellular Cd by 87%, while *copA* and *HMT* increased that by 3.2- and 3.7-folds, respectively. The strain Y8 characterized here can be a platform for exploring heavy metal genomics of the *Cellulomonas* genus.

## Materials and Methods

### Strain Isolation and Identification

Soil samples used in this study were collected from an agro-ecosystem experimental station (37°53′ N, 114°41′ E). Soil suspensions were vortexed for 60 s, followed by serial dilution and spreading onto Luria-Bertani agar medium (tryptone 10.00 g/L, yeast extract 5 g/L, NaCl_2_ 10.00 g/L, Agar 15.00 g/L) with 16 mM CdCl_2_. Plates were incubated at 30°C for 90 days, and single colonies from the plates were transferred to new LB medium plates for purification.

Genomic DNA of isolates was extracted using the PureLink Pro 96 Genomic DNA Purification Kit (Thermo Fisher Scientific, United States) following the manufacture’s instruction. The universal primers 27f and 1492r were used for 16S rRNA gene amplification (Weisburg et al., 1991). PCR products were purified after agarose gel electrophoresis and then sequenced for phylogenetic identification. Sequence alignment was performed using ClustalX (Larkin et al., 2007). DNA-DNA hybridization (DDH) and average nucleotide identity (ANI) calculation were performed using GGDC 2.1 and ANI calculator, respectively (Meier-Kolthoff et al., 2013; Jain et al., 2018).

A representative strain, namely Y8, was subjected to phenotypic characterization by BeNa Culture Collection (BNCC), Beijing, China using a VITEK 2 GP kit (Terhune, 2017). Antibiotic resistance test was performed on LB medium supplemented with antibiotics at the common working concentrations.

### Full-Genome Sequencing

High-throughput sequencing was performed for strain Y8 to obtain its complete genome. The sequencing and genome assembly methods have been reported elsewhere (Chen et al., 2019b). Briefly, the genome sequencing is completed by Genewiz (Nanjing, CN) using the Illumina HiSeq (San Diego, United States) and PacBio RS II platforms (Menlo Park, United States) according to standard protocols (Chen et al., 2019a).

### Genome Annotation

Prodigal v2.6.3 (Tennessee, United States) was used to explore coding genes following the developer’s instruction. Transfer RNAs (tRNAs) were detected in the genome using tRNAscan-SE v2.0 (Santa Cruz, United States) with default parameters (Lowe and Eddy, 1997). rRNA genes were identified by RNAmmer (Oslo, Norway) (Lagesen et al., 2007). Protein-coding genes were assigned using BLASTp against five mainstream databases including the Non-redundant Protein Database (Pruitt et al., 2005), Kyoto encyclopedia of genes and genomes (KEGG) (Kanehisa and Goto, 2000), Cluster of Orthologous Groups of proteins (KOG) (Tatusov et al., 1997), Gene Ontology (GO) (Harris et al., 2004), and Carbohydrate-Active enZYmes Database (Lombard et al., 2014). Clusters of orthologous genes (COGs) were retrieved from Y8, *C. hominis* and *C. denverensis* genomes using the OrthoFinder 1.1.8 stand-alone tool (Oxford, United Kingdom) (Emms and Kelly, 2015). Whole-genome based phylogenetic tree was constructed using Composition Vector Tree Version 3 (CVTree3)^1^ according to the online manual (Zuo et al., 2018). Circular representation of Y8’s genome was performed using Circos (Krzywinski et al., 2009), where the calculation of average G+C content and GC skew was completed using an in-house Perl v5.28 scripts.

### Cadmium Resistance Characterization

Minimum inhibitory concentration (MIC) test was conducted using the plate diluting method (Aleem et al., 2003). The MIC was defined as the lowest concentration that completely inhibited visible bacterial growth after overnight incubation (Andrews, 2001). Growth curves were determined using the method described in our previous study with minor modifications (Zheng et al., 2019; Xing et al., 2020). A Cd gradient of 0, 1, 2, 3, 4, 5, 6, and 7 mM in LB medium plates was used to test growth of strain Y8. Cell density was measured by a biophotometer (Eppendorf, Germany) at a 2-h interval until the control reached the stationary phase.

Cell morphology was observed by scanning electron microscopy (SEM). Briefly, cells were incubated overnight in LB liquid medium with or without Cd (0, 1, and 4 mM) for 4 h. Harvested cells were fixed with glutaraldehyde (2%, final conc.) overnight at 4°C. Fixative and salts were washed from the samples by centrifuging and re-suspending the pellet in Millipore^®^ water. A total of 50 μl re-suspensions were incubated in a 1.5 ml tube at room temperature for 1 h. Samples were dehydrated by soaking sequentially in ethanol solutions with five gradient concentrations of 30, 46, 63, 82, and 96% for 5 min each. After critical point drying (CPD, Quorum K850), images (FEI scanning electron microscope, HITACHI Regulus 8100) were taken following the standard instructions.

Cd bioaccumulation capacity of Y8 was determined following the method described elsewhere (Zheng et al., 2019). Briefly, the strain Y8 were cultured overnight, then transferred into 100 ml LB liquid medium with 10 μM of Cd. After 48 h incubation, cells were harvested by centrifugation and dried. The sample was digested using 8 ml of 65% HNO_3_, and dissolved in 2 ml Millipore^®^ water for Cd determination using a Zeenit 700 P atomic absorption spectrometer (Analytik Jena, Germany) equipped with a flame atomizer.

### Screening of Cadmium Resistance Genes

Candidate metal transport/resistance genes were examined genome-wide based on the genome annotation. These genes were further analyzed following the criteria of gene length, functional domains/motifs and operon organization. Known Cd resistance genes are normally with a length > 900 bp, contain common metal binding motifs like CxC, and mostly are arranged in operons and not constitutively expressed (Das et al., 2016). Domain analysis was performed using Pfam 33.1 (El-Gebali et al., 2019). Transmembrane helices in proteins were predicted using TMHMM Server v. 2.0 (Krogh et al., 2001). Phylogenetic analysis was performed using MEGA 7.0 (Kumar et al., 2016). Operons were predicted via FGENESB (Solovyev and Asaf, 2011). All the candidate genes were manually re-checked by searching them against the NCBI Nr database (Pruitt et al., 2005) and UniProt database (UniProt Consortium, 2019).

### RNA Extraction and cDNA Library Construction

Expression levels of four candidate Cd-resistance genes in strain Y8 in response to Cd stress were determined. A Cd gradient of 1, 4, and 16 mM were added directly upon inoculation (OD_600_ = 0.1), and cell samples were collected at different time points (0, 0.5, 3, 6, and 9 h) for RNA extraction. Controls were without Cd added. RNA was isolated using the MoBio microbial RNA isolation kit according to manufacturer’s instructions. Purified RNA was eluted in nuclease free water and 1 mg of RNA from each Cd treatment group was subjected to DNase treatment (30 min, 37°C). cDNA synthesis reaction was conducted with a cDNA synthesis kit (Qiagen).

### Real-Time PCR

Real-time PCR was performed in 20 μl reaction volumes containing 10 μl of the 2 × SYBR Green mastermix (ABI). The thermal conditions for PCR reactions include initial denaturation for 10 min at 95°C, followed by 40 cycles of denaturation for 15 s at 95°C and annealing for 60 s at 60°C for gene amplifications. Real-time PCR was carried out in an ABI 7000 PCR system and melting curve analysis was performed within the temperature range of 67–95°C. The Ct values were determined, and *thyA* gene was used as an internal reference (Yan et al., 2019).

### Chemical Synthesis of Candidate Genes

Chemical synthesis of all candidate gene was completed by Sangon Shanghai, China. The vector pTR modified based on pUC19 (Li et al., 2020) was employed to carry the four potential resistance genes. The pTR vector contains a tobacco plastid 16S ribosomal RNA gene (P16S) promoter, multiple cloning sites and a *rrnB* T1 terminator (BBa_B0010) located between the restriction endonuclease (RE) site *Hin*dIII and *Eco*RI. All selected genes were reverse transcript to 5′–3′ direction. For sequences with locally excessive G+C content (>90%), the codons were optimized to better translate them in *E. coli*. Meanwhile, suitable RE sites were added to both ends of all the sequences. All of recombinants were enzymatically digested according to their designed RE sites, and sequenced to double-check the quality.

### Functional Verification of Candidate Cadmium Resistance Genes

Four recombinants containing the synthesized gene, pTR-*zntAY8*, pTR-*copAY8*, pTR-*HMTY8*, and pTR-*czcDY8*, were subjected to functional tests via heterologous expression in *E. coli* DH5α (F-, Φ80, lacZ, ΔM15, Δ *lacU*169, recA1, *endA1*, *hsdR17*, *supE44*, *thi-1*, *gyrA*, *relA1*, *λpir*). Cd-sensitive *E. coli* RW 3110 (F-, *λ^–^*, *IN(rrnD-rrnE)1*, *zntA1(CdS,ZnS)::kan*, *rph-1*) was employed as host cells for further functional verification (Li et al., 2020). The plasmid pTR without any candidate genes was transformed into RW 3110 to generate a negative control. The threshold Cd concentration used to test transformants for Cd resistance is 0.3 mM, which was determined in our previous study (Li et al., 2020). The grow curve of all transformants was tested as follows. Briefly, transformant cells were incubated overnight. Aliquots of cells were then inoculated into 100 ml LB liquid medium supplied with Cd with a starting OD_600_ of 0.1, and incubated at 37°C. The optical density at 600 nm was measured by spectrophotometer every hour for 12 h.

Metal bioaccumulation assay was conducted according to our previous study with minor modifications (Xing et al., 2020). Four transformants and the control were cultured overnight. Five ml of each was then inoculated into 100 ml LB liquid medium (10 μM, Cd) and incubated for 6 h. The cells were collected by centrifugation at 4,000 × g, rinsed triple times using water rigorously and subsequently dried, weighed, and digested in 7 ml of 65% HNO_3_. The digested mixture was dissolved in 2 ml Millipores water and the metal content was measured using ICP-MS. Certified reference material laver (GWB10023, certified by the Institute of Geophysical and Geochemical Exploration, China) was used as a standard reference material for Cd, Ni, Cu, and Zn determination.

### Data Analysis and Availability

Statistical analysis was performed with SPSS (IBM, Armonk, United States) and Office suits (Microsoft, Redmond, United States). Full genome of strain Y8 can be accessed via the accession number CP041203.1 in the NCBI database.

## Results

### The Morphological and Physiological Features of *Cellulomonas* Strain Y8

Strain Y8 was the only isolate identified in this study that could form colonies on solid LB plate supplied with 16 mM of Cd after a 90-day incubation. Cells of Y8 were seen to be aerobic, rod-shaped and Gram-positive. After 48 h of incubation on solid LB agar plate at 30°C, the colonies produced by this bacterium (0.5–1 mm in diameter) were smooth, opaque, moist, and pale yellow in color.

A BLAST search of Y8’s 16S rRNA gene showed a 99.57% similarity to that of *C. pakistanensis* NCCP-11, 99.13% to *C. hominis* JCM 12133, and 98.37% to *C. denverensis* W6929, suggesting that Y8 is a member of *Cellulomonas*.

Basic biochemical characteristics of strain Y8 showed that Y8 had an optimum growth at 28–32°C, and was able to ferment a wide variety of sugars like D-cellobiose, D-glucose, D-maltose, and D-mannose but not D-tagatose (Supplementary Table 1). Y8 was resistant (μg/ml) to ampicillin (100), apramycin (50), spectinomycin (50), gentamicin (50), and kanamycin (50), and sensitive (μg/ml) to chloramphenicol (25) and erythromycin (100).

DDH and ANI were used as minimal criteria for the identification of novel species here (Chun et al., 2018). The level of DDH between strain Y8 and *C. pakistanensis*, *C. hominis*, and *C. denverensis* were 52.5, 39.5, and 22.3%, respectively, which were below the 70% cutoff value suggested for species identification. ANI was estimated to be 93.79% between strain Y8 and *C. pakistanensis*, 84.25% between strain Y8 and *C. hominis* and 81.47% between strain Y8 and *C. denverensis*.

### Full Genome of Strain Y8

We obtained the high-quality full genome of strain Y8 of 4,475,991 bp in this study. Y8’s genome has a G+C content of 75.35%, and contains 4,074 coding sequences (CDSs) with an average length of 982 bp.

A whole-genome based phylogenetic tree was constructed (Supplementary Figure 1). *C. hominis*, *C. denverensis*, and Y8 were assigned to orthologous groups (orthogroups) of Y8 using OrthoFinder. A total of 10,077 protein-coding genes (90.8% of the total) were assigned to 3,068 orthogroups, of which 2,224 included representatives from all three genomes and 1,715 were single-copy orthogroups. Y8 shared 2,789 orthologs with *C. hominis* and 2,602 with *C. denverensis*.

### Cadmium Resistance of Strain Y8

**FIGURE 1:**
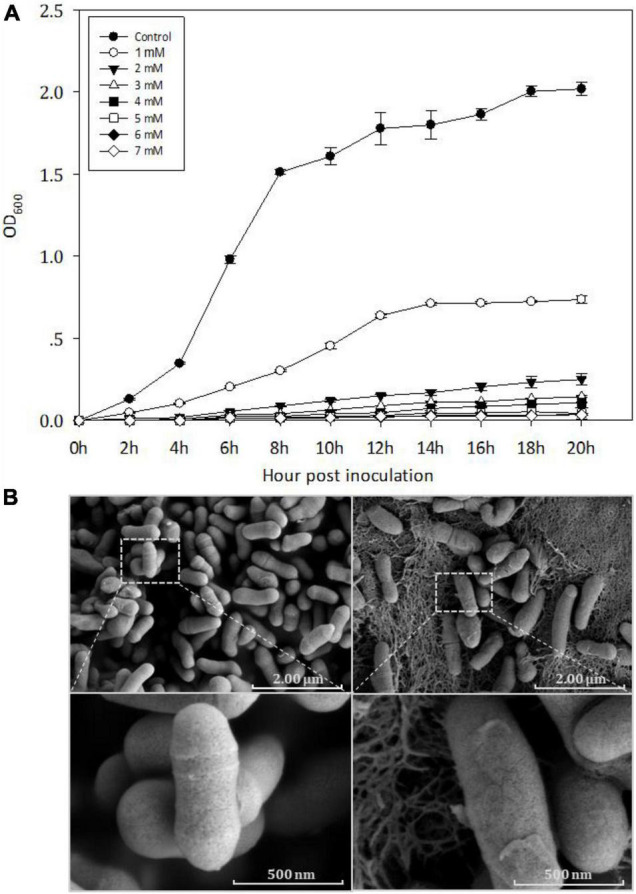
**(A)** Growth curves of Y8 exposed to Cd of different concentrations; **(B)** scanning electron microscope (SEM) images of Y8’s cell morphology without (left) and with Cd (right, 4 mM).

The MIC of Cd for Y8 was found to be 5 mM (Figure 1A). At 4 mM Cd, Y8’s cells appear in an irregular rod shape with a smooth surface based on the SEM imaging results (Figure 1B), while a thickened cell wall was observed at all Cd treatments. Cd bioaccumulation assay showed that Y8 had an adsorption capacity of 15.80 mg/g Cd when treated with 1 mM CdCl_2_, and 66.54 mg/g when treated with 4 mM CdCl_2_.

### Cadmium Resistance Genes in Y8’s Genome

**FIGURE 2:**
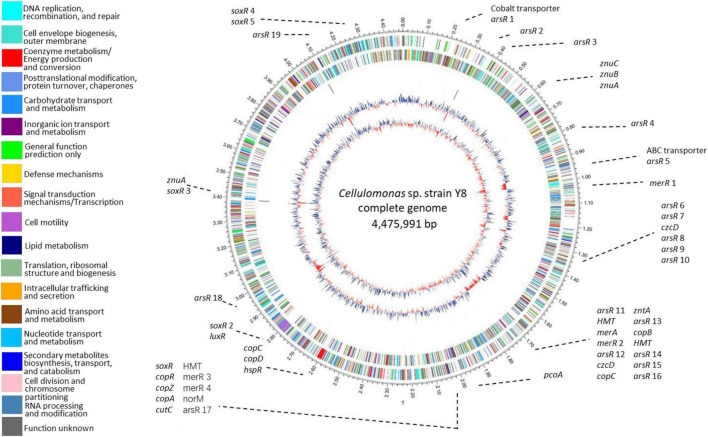
Circular representation of Y8’s genome. Circles display from the inside outwards, (1) GC-skew (G-C/G+C ratio) using a 999 bp window; (2) GC-content using a 999 bp window; (3) ncRNA genes on the minus strand; (4) ncRNA genes on the plus strand; (5) COG assignments for predicted CDSs on the minus strand; (6) COG assignments for predicted CDSs on the plus strand; (7) scale in Mb.

We identified 54 metal-resistance related genes throughout Y8’s genome (Figure 2 and Supplementary Table 2). Four potential Cd transporting genes, namely *zntAY8*, *copAY8*, *HMTY8*, and *czcDY8*, were chosen for functional verification. Flanking genes in the operons and domains of *zntAY8*, *copAY8*, *HMTY8*, and *czcDY8* were analyzed (Supplementary Figure 2 and Supplementary Table 3). Briefly, the *znt* operon carrying *zntAY8* (2,319 bp) comprises three genes including *zntAY8*, a hypothetical gene and an *arsR*-family gene. ZntAY8 shares a sequence similarity of 39.29 and 39.05% with ZntA from *E. coli* K12 and ZntA from *Shigella sonnei* strain Ss046, respectively. The operon carrying *copAY8* is 3,484 bp and comprises four genes including a hypothetical gene, a repressor gene, a Cu chaperone gene *copZ* and *copAY8*. The protein CopAY8 shares a sequence similarity of 47.54 and 40.02% with CopA from *E. coli* K-12 and *B. subtilis* strain 168, respectively. The operon carrying *HMTY8* (3,628 bp) consists of four genes including a thioredoxin reductase gene, *HMTY8*, an *arsR*-family gene and a hypothetical gene. Sequence similarity between HMTY8 and ACR3 from *Corynebacterium glutamicum* strain ATCC 13032 is 33.67%. The operon carrying *czcDY8* (2,728 bp) contains four genes including an *arsR*-family gene, *czcDY8*, *STE14* encoding a putative protein-S-isoprenylcysteine methyltransferase, and *ompR*. CzcDY8 shares a protein similarity of 33.67% with CzcD from *B. subtilis* strain 168 and 39.05% with CzcD from *C. metallidurans* strain ATCC 43123.

**FIGURE 3:**
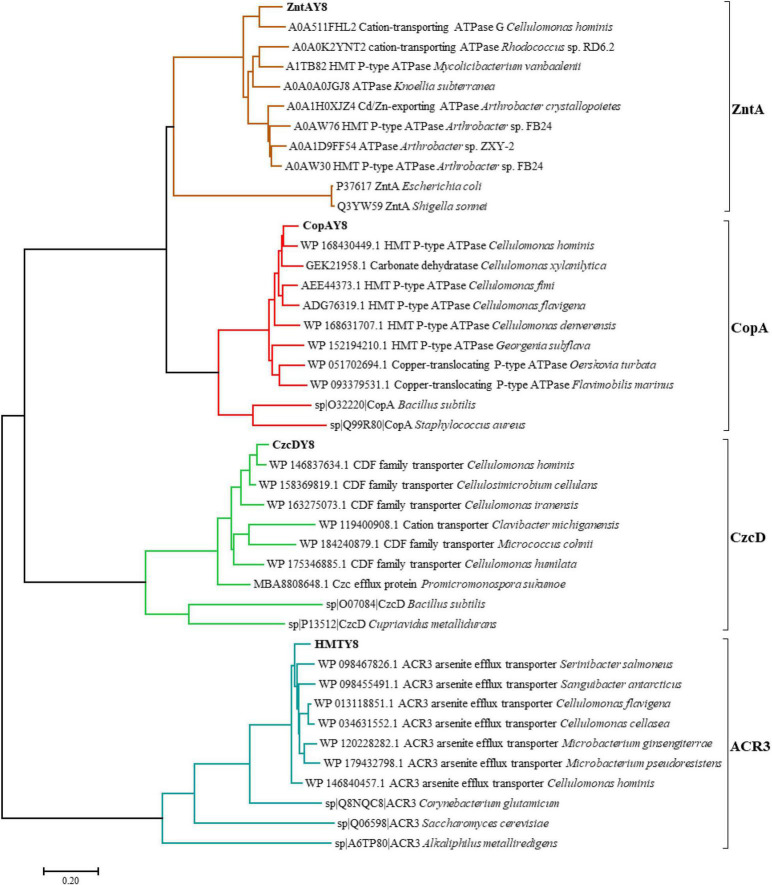
A phylogenetic tree showing the protein sequence similarity of candidate metal resistance genes in strain Y8’s genome and homologs. The tree was constructed using the Maximum Likelihood method within MEGA 7.0 (Kumar et al., 2016). Bootstrap values were estimated from 1,000 replicates. Sequence alignment was performed using ClustalX (Larkin et al., 2007).

A phylogenetic tree (Figure 3) was constructed to explore the evolutionary relationships between ZntAY8, CopAY8, HMTY8, CzcDY8, and their homologous proteins.

### Time- and Dose-Dependent Expression of Selected Genes in Strain Y8

**FIGURE 4:**
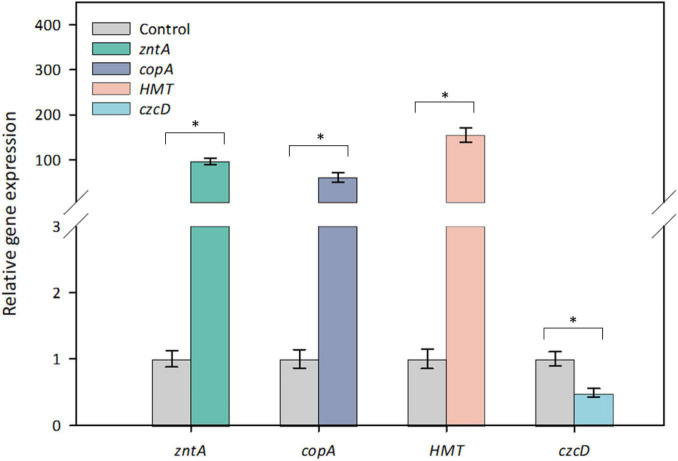
Relative gene expression of the tested genes of strain Y8 in response to 4 mM Cd. Differences between paired values with 3 experiments that are statistically significant as determined by *t*-test are denoted as follows: * *p* < 0.01.

**FIGURE 5:**
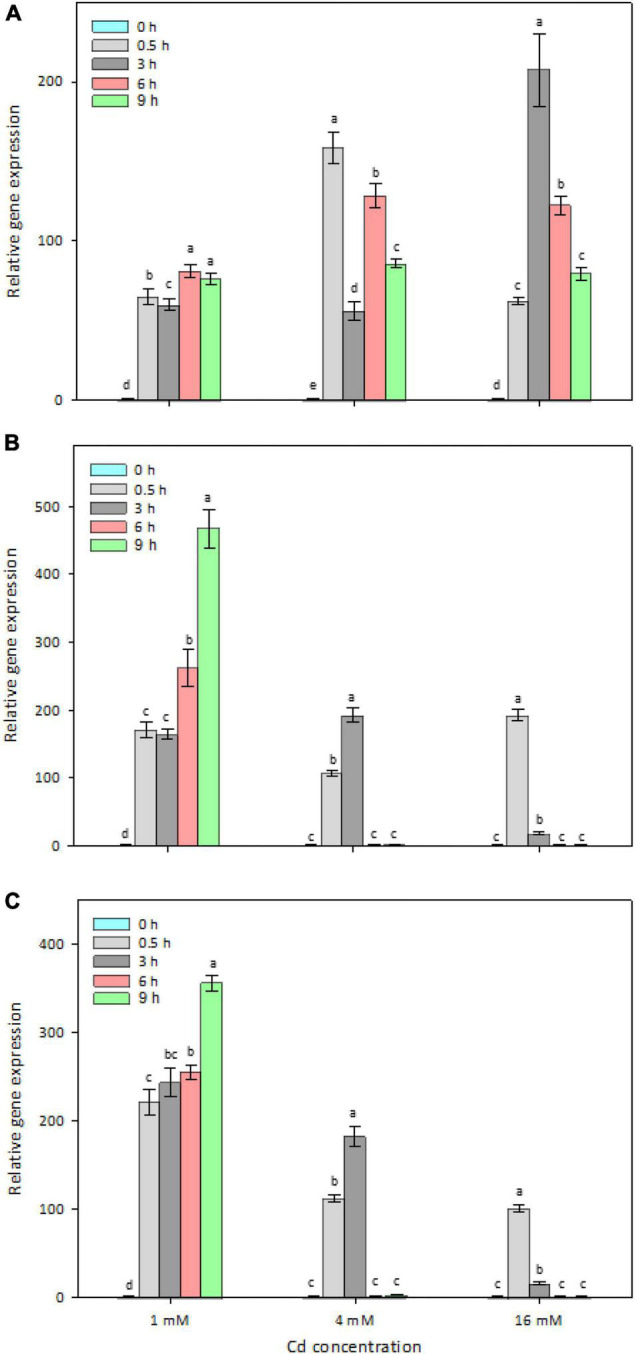
Time-dependent and Cd-level-dependent responses of the Cd-inducible metal resistance genes. **(A)** *zntAY8*; **(B)** *copAY8*; **(C)** *HMTY8*.

To determine whether the four candidate genes are Cd-inducible in strain Y8, their *in vivo* expression levels were determined by RT-qPCR (Figure 4). After 6 h treatment with 4 mM CdCl_2_, *zntAY8*, *copAY8*, and *HMTY8* were significantly upregulated, while *czcDY8* was slightly but significantly downregulated (Figure 4). In order to gain a more comprehensive understanding of their roles in the response of Y8 to Cd stress, the time-course expression of *zntAY8*, *copAY8*, and *HMTY8* under different Cd concentrations were further quantified. As shown in Figure 5, all the three tested genes were significantly induced at 1 mM Cd at all-time points, while only the gene *zntAY8* responded constantly at higher Cd concentrations. Expression levels of *copAY8* and *HMTY8* decreased over time substantially at higher Cd concentrations (Figures 5B,C).

### Functional Verification of the Candidate Cadmium Resistant Genes in *Escherichia coli* Strain RW 3110

The four genes, *zntAY8*, *copAY8*, *HMTY8*, and *czcDY8*, were heterologously expressed in Cd-sensitive *E. coli*. Quality of the recombinant plasmids (pTR-*zntAY8*, pTR-*copAY8*, pTR-*HMTY8*, and pTR-*czcDY8*) were checked through double enzyme digestion detection (Supplementary Figure 3), and double-checked by Sanger sequencing before being transformed into the hosts.

**FIGURE 6:**
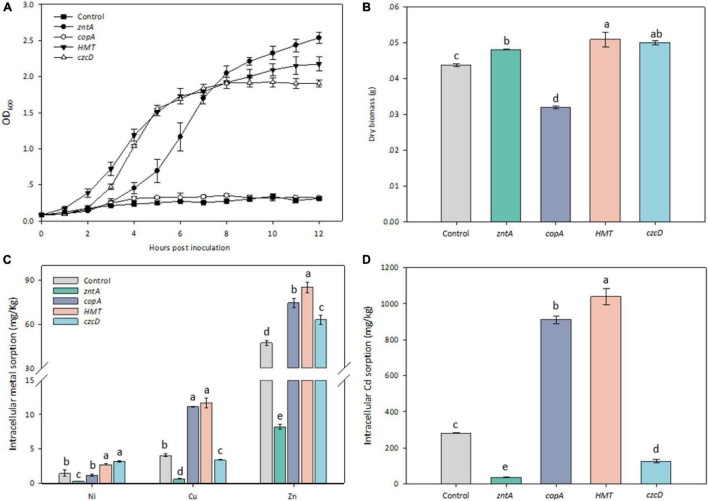
Phenotypic response of Cd-sensitive strain harboring the tested metal resistance genes. **(A)** Growth curves of transformants harboring the tested resistance genes under 0.3 mM Cd; **(B)** dry biomass of transformants harboring the tested resistance genes; **(C)** cellular accumulation of Ni, Cu, and Zn by transformants harboring the tested resistance genes; **(D)** cellular accumulation of Cd by transformants harboring the tested resistance genes.

Growth curves of *E. coli* RW 3110 overexpressed with and without the recombinant plasmids were determined under 0.3 mM Cd stress. The threshold Cd concentration used to test the transformants referred to our previous study (Li et al., 2020). The growth rates of RW 3110 with all the tested genes except for *copAY8* were considerably higher than the control (Figure 6A). The transformants *zntAY8*, *HMTY8*, and *czcDY8* reached the exponential phase at 2–3 h.

After 6 h culture, dry biomass of all transformed strains was significantly different from that of the control (Figure 6B). The biomass of RW 3110 with *zntAY8*, *HMTY8*, and *czcDY8* was around 10% higher, while RW 3110 with *copAY8* was lower than the control. Cd sorption in RW 3110 with *copAY8* and *HMTY8* increased by 3.22 and 3.68 folds, while that in RW 3110 with *zntAY8* and *czcDY8* decreased by 86 and 53.3% compared with the control, respectively (Figure 6D). Uptake of Ni, Cu, and Zn by transformed strains in Cd-containing medium was also determined (Figure 6C). Results showed that Zn accumulation in RW 3110 with *copAY8*, *HMTY8*, and *czcDY8* increased by 34–80%, while that of RW 3110 with *zntAY8* decreased by 87.3%. Cu and Ni accumulation in all transformants showed a similar trend to that for Zn.

## Discussion

Cadmium is an extremely toxic element, due to its mutagenic effect (Jin et al., 2003) and ability to cause indirect formation of reactive oxygen species (ROS) (Waisberg et al., 2003). Cd tolerance of bacteria varies among species and can be partially reflected in their MIC. The Cd MIC of strain Y8 (5 mM) is much higher than common strains, such as *S. aureus* (<0.16 mM on agar plates) (Rosdahl and Rosendal, 1980), as well as some Cd resistant isolates including *Pseudomonas stutzeri* (0.6 mM on LB plates) (Deb et al., 2013), *Pseudomonas* sp. TeU (0.5 mM on LB plates) (Chien et al., 2011) and *Lactococcus lactis* (1.78 mM on MRS agar plates) (Sheng et al., 2016). While strain Y8 lacks any plasmids for encoding commonly known resistance genetic systems, it is supposed that some chromosome-borne genes are responsible for its Cd resistance.

Bacterial exposure to extreme Cd stress can normally cause a sharp drop in growth rate and a series of morphological changes including cell shrinkage and even the complete loss of cell structure (Hou et al., 2015; Khan et al., 2015; Sheng et al., 2016; Huang et al., 2018). The strain Y8 was significantly inhibited in growth by 4 mM CdCl_2_ exposure (Figure 1A), whereas no obvious change was observed on cell morphology (Figure 1B). Meanwhile, Y8 cells produced a large amount of extracellular reticulum structure under Cd stress (Figure 1B). These reticulate substances were supposed to be extracellular polymer substances (EPS), which are generally secreted in the form of biofilm (Florentin et al., 2012). Several studies had reported that members of *Cellulomonas* genus such as *C. flavigena*, *C. uda*, and *C. fimi* can form biofilms (McIntosh et al., 2005; Young et al., 2012), which is a curdlan-type matrix. Genome annotation of the strain Y8 here detected multiple copies of biofilm formation related gene such as *wcaA*, *wcaG*, and glycosyltransferases (Zheng et al., 2018; Oehme et al., 2019). Previous studies indicated that EPS can immobilize metals through ionizable groups such as -OH, -NH, and -COOH (Shen et al., 2018; Xie et al., 2020), to reduce the toxicity of heavy metals. We speculated that the observed reticulate substances are EPS and may play a role in Cd resistance of strain Y8, yet further experimental evidences are needed to classify.

An important feature of Y8’s genome is its high GC content (Figure 2). Strain Y8 has a GC content of more than 75%, which is beyond the currently known range of genomic GC skew (Romiguier and Roux, 2017). Genomic base composition variation is shaped by various evolutionary events, leading to differential biological functions (Wu et al., 2012). It is generally thought that high-GC content is associated with a lower mutation rate under high selective pressure. Our recent study has documented that prokaryotic extremophiles commonly possess high-GC genomes, such as the Cu-resistant *Cupriavidus* strains with an average GC content of 66.2%, the multi-metal resistant *Thiobacillus* strains with an average GC content of 62.6%, the radiation and/or metal tolerant *Deinococcus* strains with an average GC content of 67.3%, and the Zn-resistant *Comamonas* spp. with a GC content of 61.3–61.5% (Chen et al., 2019a,b). A high GC content may help strain Y8 in maintaining its key genetic elements under extreme metal stress which may cause a high rate of DNA damage.

The diversity of genetic elements related to metal stress within Y8’s genome is vast. We identified more than 50 genes for dedicated metal stress response (Figure 2 and Supplementary Table 2), accounting for 1.2% of the total genes. This frequency of metal-associated genes is comparable to that of the microbial metagenome from metal mine tailings of extremely abundant heavy metals (Li et al., 2015). Similar to *C. metallidurans* strain CH34, a model bacterium for metal resistance study, a variety of metal efflux systems were detected in Y8’s genome, including genes of the P-type ATPase, ABC transporter, and CDF transporter families (Nies). Nevertheless, only two genes, *zntA* and *czcD*, were thought to be dedicated to Cd resistance. Though versatile genes for multi-metal resistance have been reported (Solovieva and Entian, 2004; Steunou et al., 2020), it is unknown whether the remaining metal resistance genes in Y8’s genome, like the candidate *copA* and *HMT*, play a potential role in Cd resistance. Surprisingly, all the four genes were Cd-inducible and three of them restored Cd resistance of the Cd sensitive strain heterologously, as discussed below.

It is worth noting that most of Y8’s metal resistance genes are organized into operons. Operons are clusters of adjacent genes encoding for proteins with related roles, which provides an efficient mechanism to coordinate the expression of neighboring genes (Sáenz-Lahoya et al., 2019). Roles of the regulator genes in the operons merit further investigation, since more than 20 copies of ArsR-family regulators were identified in Y8’s genome. Meanwhile, some unknown coding regions were annotated as structural genes of the detected operons of *znt, cop*, *HMT*, and *czc*, and some of them have overlap regions with main genes (Supplementary Figure 2). A study revealed that occurrence of overlapping gene pairs is associated with tight translational coupling (Huber et al., 2019). Basically, organization of these resistance genes in operons may enable strain Y8 a strong ability of rapid transcriptional response to metal stresses.

Modern omics tool has revealed a variety of basic metabolic pathways involved in metal resistance (Sheng et al., 2016; Isarankura-Na-Ayudhya et al., 2018; Alviz-Gazitua et al., 2019), and for either prokaryotic or eukaryotic cells possession of specific Cd resistance genes/operons is essential for Cd resistance (Intorne et al., 2012; Schwager et al., 2012; Chaoprasid et al., 2015; Zhang et al., 2015). A genome-wide annotation of strain Y8 led to the identification of 54 heavy metal-related genes (Figure 2), including potential metal resistance genes homologous to *zntA* of *E. coli*, *czcD* of *C. metallidurans*, *copAB of Legionella pneumophila* (Purohit et al., 2018), *cutC* of *Enterococcus faecalis* (Latorre et al., 2011), *znuA* of *E. coli* (Patzer and Hantke, 1998), etc. It is generally thought that most of metal transporters (Dutta et al., 2007; Smith et al., 2014) as well as regulators (Brocklehurst et al., 2003; Radford et al., 2003) are relatively specific. While it was supposed that these annotated metal resistance genes, particularly *copA*, may be dedicated for a specific metal, our results showed that at least the four tested non-Cd-specific genes responded collectively to Cd stress, which was implied by their Cd-inducible expression and Cd resistance function in *E. coli* (Figures 4–6).

The involvement of *zntAY8* and *czcDY8* in Y8’s Cd stress response (Figures 4, 5A) may be not surprising, considering that *EczntA* is responsible for specific resistance to both Zn and Cd (Rensing et al., 1997), and the *czc* system was a well-known Cd resistance determinant (Hassan et al., 1999). *zntAY8* was shown to be effective in enhancing Cd resistance of *E. coli* RW 3110 heterologously, probably as a potent multi-purpose metal exporter which was implied by the sharp reduction in *E. coli*’s Cd/Ni/Zn/Cu sorption (Figures 6B,D). With typical metal binding motifs as well as the ATPase binding site, the *zntAY8* gene is phylogenetically close to P-type ATPase genes, like the typical Cd resistance genes *cadA* and *czcP* that can mediate the extrusion of metals including Cd from cytoplasm by hydrolysis of ATP (Lee et al., 2001; Smith et al., 2014). The *czcDY8* gene was seen to be a cation diffusion facilitator. *czcD* was previously found to be part of the high-level metal resistance system *czc* that mediates the efflux of Co, Zn, and Cd ions (Munkelt et al., 2004). Different from the known czc system, two copies of *czcDY8* were detected in Y8’s genome with no *czcCBA* flanked, implying that the *czcDY8* may function independently. The function of *copAY8* and *HMTY8* seems unusual here, which both increased intracellular Cd accumulation (Figure 6D). To our knowledge, *copA* is specific for Cu translocating and resistance (Giachino and Waldron, 2020), although two studies have reported the Cd-inducible *copA* variants (Toes et al., 2008; Steunou et al., 2020). Moreover, the ACR3 gene, the closest homolog to *HMTY8*, has been rarely reported to play a role in Cd resistance (Markowska et al., 2015). Phylogenetic analysis indicated that closest homologs of these four genes are all from the genus of *Cellulomonas* (Figure 3), which is consistent with the previous viewpoint that heavy metal transporters are mostly evolving via vertical descent (Li et al., 2015). Considering that none of their homologs from this genus have been reported in terms of a role in Cd resistance, we speculate that *zntAY8*, *copAY8*, *HMTY8*, and *czcDY8* are novel metal resistance genes playing a role in Cd resistance of the genus *Cellulomonas.*

Determination of Cd-induced *in vivo* expression of *zntAY8*, *copAY8*, and *HMTY8* indicated that their response to Cd stress in Y8 was dose- and time-dependent (Figure 5). The expression of heavy metal transport systems is normally controlled at the level of transcription in order to minimize the associated metabolic burden to the host (Hynninen, 2010; Alviz-Gazitua et al., 2019). From this perspective, it is unwise for bacteria to express multiple transporters simultaneously, especially under severe Cd stress. Our results imply that *copAY8* and *HMTY8* seems to favor a low Cd stress while *zntAY8* favors a high Cd stress of up to 16 mM (Figure 5). In combination with the results of their roles in Cd accumulation (Figure 6D), the dose- and time-dependent expression of the three genes may indicate that strain Y8 may recruit a mechanism for Cd sequestration by *copAY8* and *HMTY8* under minor Cd stress which increases intracellular Cd, and trigger a mechanism for Cd exporting by *zntAY8* under sever Cd stress. Such dose-dependent mechanism for metal resistance has been inferred by cellular Cu homeostasis. Recruiting different genetic pathways for coping with high levels of metal stress has also been reported for Cu and Zn in eukaryotic cells. For example, tripeptide glutathione (GSH) is heavily produced for Cu excretion when Cu stress is high, and metallothioneins increased when Zn is absorbed in a large quantity in mammalian cells (Bertinato and L’Abbé, 2004).

Though our current results showed that the four genes that function in Cd resistance can promote either Cd intake or export and also to some extent play a role in Ni, Cu, and Zn trafficking (Figures 6C,D), more experimental evidences are needed to describe the process of metal transport as well as their affinity to metals. Their intracellular expression regulation under Cd stress remains unknown, considering that a large number of *arsR/merR* family regulator genes were detected in Y8’s genome. Available evidence allows us to conclude that *zntAY8*, *copAY8*, *HMTY8*, and *czcDY8* are novel metal resistance genes of the genus *Cellulomonas*, and they respond to Cd stress collectively in strain Y8. Meanwhile, ZntAY8 seems to be a strong Cd/Ni/Cu/Zn exporter that can substantially improve the host’s growth under metal stress.

## Data Availability Statement

The datasets presented in this study can be found in online repositories. The names of the repository/repositories and accession number(s) can be found below: https://www.ncbi.nlm.nih.gov/genbank/, CP041203.1.

## Author Contributions

XL initiated the concept and designed the experiment. JC, WL, and XZ performed the molecular experiments. LW and XL analyzed the genomic data. JC, XL, and LW draft the manuscript. All authors revised the manuscript and approved the submission.

## Conflict of Interest

The authors declare that the research was conducted in the absence of any commercial or financial relationships that could be construed as a potential conflict of interest.

## Publisher’s Note

All claims expressed in this article are solely those of the authors and do not necessarily represent those of their affiliated organizations, or those of the publisher, the editors and the reviewers. Any product that may be evaluated in this article, or claim that may be made by its manufacturer, is not guaranteed or endorsed by the publisher.

## Footnotes

1
http://cvtree.online/v3/cvtree/
