# Supplementary material for: Genomic Insights Into Cadmium Resistance of a Newly Isolated, Plasmid-Free Cellulomonas sp. Strain Y8
Source: Front Microbiol. 2022 Jan 28;12:784575. doi: 10.3389/fmicb.2021.784575 (PMC8832061; doi:10.3389/fmicb.2021.784575)
Supplement: Supplementary file 1 [file Data_Sheet_1.DOCX]

**Genomic insights into cadmium resistance of a newly-isolated, plasmid-free *Cellulomonas* sp. strain Y8**

Jinghao Chen^1,2†^, Likun Wang^1†^, Wenjun Li^1,2^, Xin Zheng^1^ and Xiaofang Li^1*^

^1^ Hebei Key Laboratory of soil Ecology, Center for Agricultural Resources Research, Institute of Genetics and Developmental Biology, Chinese Academy of Sciences, Shijiazhuang 050021, China

^2^ University of Chinese Academy of Sciences, Beijing 100049, China

^*^Correspondence to XL, Email: xfli@sjziam.ac.cn; Postal address: No. 286 Huaizhong Rd, Shijiazhuang, China; Tel: 86 311 85822874; Fax 86 311 85815093

^†^J.C. and L.W. contributed equally to this work.

Running title: Cadmium resistance genomics of *Cellulomonas* sp. strain Y8

**Acknowledgments**

This work was supported by the Hebei Science Fund for Distinguished Young Scholars (No. D2018503005), the National Natural Science Foundation of China (No. 41877414) and the National Key Research and Development Program of China (No.2018YFD0800306).

**Competing interests**

We declare no competing financial interests.

**Contributions**

X.L. initiated the concept and designed the experiment. J.C., W.L., L.C. and X.Z. performed the molecular experiments. L.W. and X.L. analyzed the genomic data. J.C, X.L. and L.W. draft the manuscript. All authors revised the manuscript and approved the submission.

**Suppl. Table S1** Phenotypic characteristics of strain Y8 isolated in this study

| Category | Test | Result |
| --- | --- | --- |
| Basic characteristics | Motility | + |
|  | Temperature range (℃) (optimum) | 10–37 (28–32) |
| Assimilation | L-lactate | - |
|  | L-malate | - |
|  | L-histidine | - |
| Acid production from: | D-cellobiose | + |
|  | D-glucose | + |
|  | D-maltose | + |
|  | D-mannose | + |
|  | Panatinose | + |
|  | Saccharose/Sucrose | + |
|  | D-tagatose | - |
| 0 | D-trehalose | + |
|  | Alpha-glucosidase | + |
|  | Adonitol | - |
|  | L-Arabitol | - |
|  | D-mannitol | + |
|  | D-sorbitol | - |
| Enzyme activity | L-Pyrrolydonyl- arylamidase | + |
|  | Ala-Phe-Pro-arylamidase | + |
|  | Beta-Galactosidase | + |
|  | Beta-N-acetyl-glucosaminidase | + |
|  | Glutamyl arylamidase pNA | - |
|  | Gamma-glutamyl-Transferase | - |
|  | BETA-xylosidase | + |
|  | BETA-Alanine arylamidase pNA | - |
|  | L-Proline arylamidase | + |
|  | Lipase | - |
|  | Tyrosine arylamidase | + |
|  | Urease | + |
|  | Beta-N-acetyl galactosaminidase | - |
|  | Phosphatase | - |
|  | Glycine arylamidase | - |
|  | Ornithine decarboxylase | - |
|  | Lysine decarboxylase | - |
|  | Glu-Gly-Arg-arylamidase | - |

**Suppl. Figure S1** A whole genome-based phylogenetic tree showing evolutional relationship between strain Y8 and close relatives

**Suppl. Table S2** Genomic annotation of potential metal resistance genes in strain Y8’s genome

**
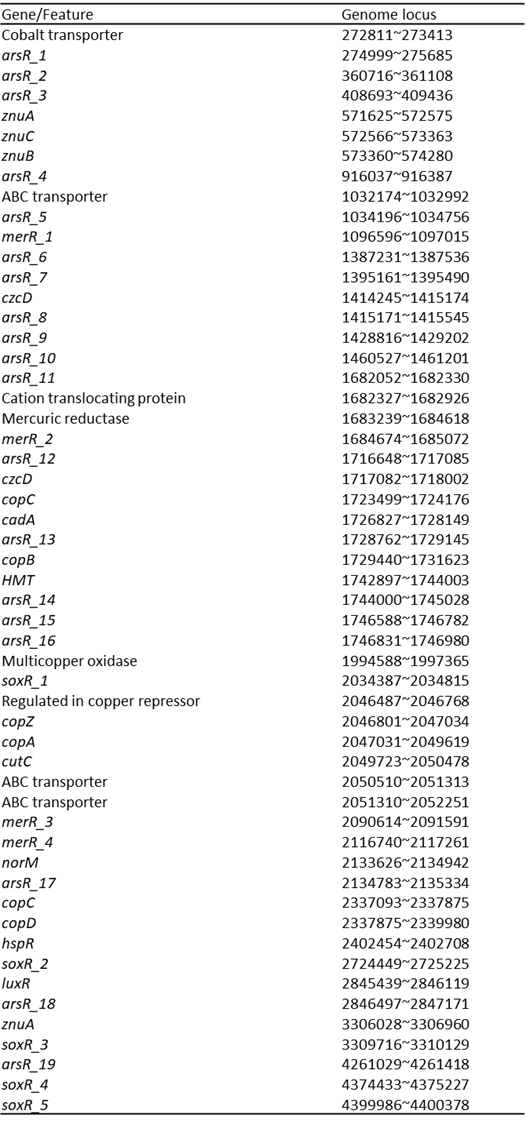
**


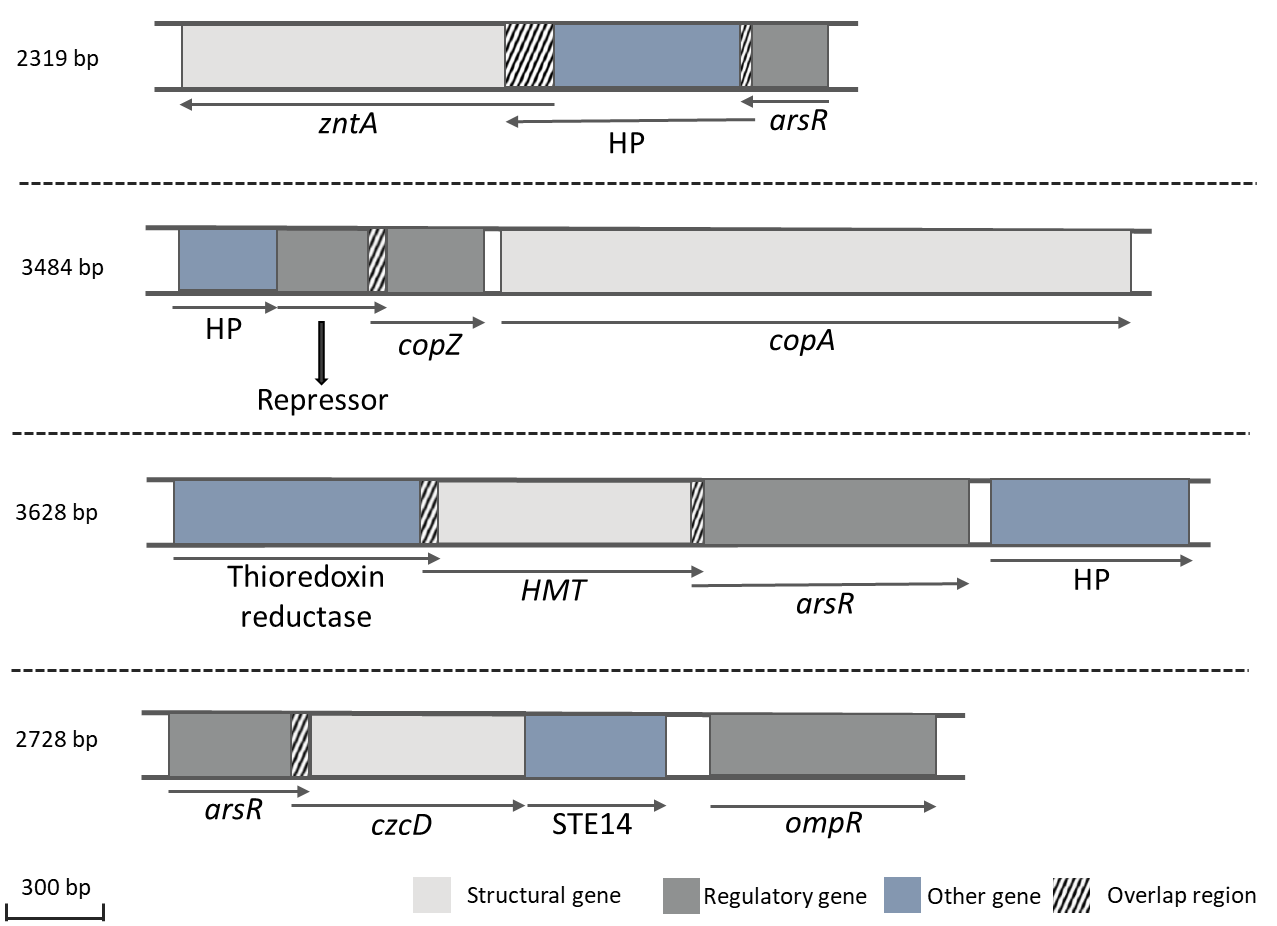


**Suppl. Figure S2** Physical maps of operons containing the four tested genes in strain Y8’s genome. HP, hypothetical protein. HMT, heavy metal transporter

**Suppl. Table S3** Sequence features of the four candidate metal-resistance genes subjected to functional characterizations in this study

| Proteins | Length (aa) | Transmembrane (position) | | | | Conserved residues/motifs | | | | | |
| --- | --- | --- | --- | --- | --- | --- | --- | --- | --- | --- | --- |
|  |  | TMs number | Typical TM regions | | |  |  |  |  |  |  |
| ZntA | 440 | 3 | TM1  (48-70) | TM2  (85-107) | TM3  (381-403) | S^89^PC | D^133^KTGT | H^154^ | P^301^EDK | G^321^DGVND | L^360^HHL |
| CopA | 889 | 8 | TM5  (465-487) | TM6  (497-519) | TM7  (843-862) | C^29^ASC | H^126^AGH | C^479^PC | D^523^KTGT | G^757^DGVND | / |
| HMT | 368 | 10 | TM1  (21-40) | TM2  (50-72) | TM3  (88-110) | Sodium Bile acid symporter domain (60-256) | / | / | / | / | / |
| CzcD | 306 | 6 | TM2  (51-69) | TM3  (81-109) | TM5  (150-161) | H^3^GHDH | H^236^DLH | / | / | / | / |


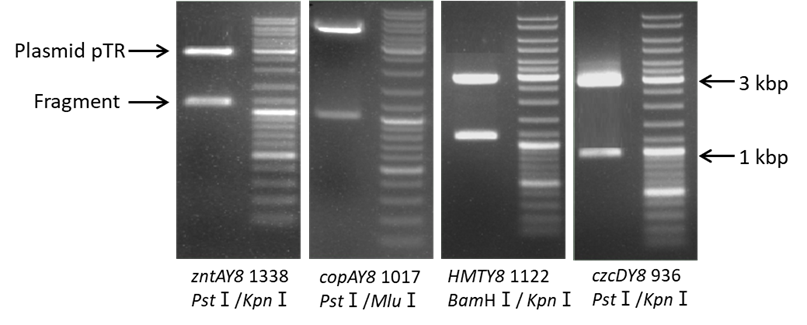
 **Suppl. Figure S3** Double enzyme digestion check of the four recombinant plasmids
